# A Structural Study on the *Listeria Monocytogenes* Internalin A—Human E-cadherin Interaction: A Molecular Tool to Investigate the Effects of Missense Mutations

**DOI:** 10.3390/toxins12010060

**Published:** 2020-01-20

**Authors:** Luca Dellafiora, Virginia Filipello, Chiara Dall’Asta, Guido Finazzi, Gianni Galaverna, Marina Nadia Losio

**Affiliations:** 1 Department of Food and Drug, University of Parma, Parco Area delle Scienze 27/A, 43124 Parma, Italy; chiara.dallasta@unipr.it (C.D.); gianni.galaverna@unipr.it (G.G.); 2 Department of Food Control, Istituto Zooprofilattico Sperimentale della Lombardia e dell’Emilia Romagna, Via A. Bianchi 9, 25124 Brescia, Italy; guido.finazzi@izsler.it (G.F.); marinanadia.losio@izsler.it (M.N.L.)

**Keywords:** *Listeria monocytogenes*, in silico modeling, internalin A, E-cadherin, cell invasion

## Abstract

*Listeria monocytogenes* is a widespread foodborne pathogen of high concern and internalin A is an important virulence factor that mediates cell invasion upon the interaction with the host protein E-cadherin. Nonsense mutations of internalin A are known to reduce virulence. Although missense mutations are largely overlooked, they need to be investigated in respect to their effects in cell invasion processes. This work presented a computational workflow to early characterize internalin A missense mutations. The method reliably estimated the effects of a set of engineered missense mutations in terms of their effects on internalin A–E-cadherin interaction. Then, the effects of mutations of an internalin A variant from a *L. monocytogenes* isolate were calculated. Mutations showed impairing effects on complex stability providing a mechanistic explanation of the low cells invasion capacity previously observed. Overall, our results provided a rational approach to explain the effects of internalin A missense mutations. Moreover, our findings highlighted that the strength of interaction may not directly relate to the cell invasion capacity reflecting the non-exclusive role of internalin A in determining the virulence of *L. monocytogenes*. The workflow could be extended to other virulence factors providing a promising platform to support a better molecular understanding of *L. monocytogenes* epidemiology.

## 1. Introduction

*Listeria monocytogenes* is a widespread, gram-positive, opportunistic intracellular pathogen with the capacity to actively invade and multiply within a broad range of animal cells [1]. *L. monocytogenes* is the etiologic agent of listeriosis, a foodborne disease affecting both humans and animals (mainly ruminants) [2,3]. Concerning human cases, listeriosis generally affects people with an altered immune system and it is characterized by low incidence and high case-fatality rate (up to 30%), with a great burden of disease in terms of impact on the individual, public health costs and food production losses [4,5]. Typically, *L. monocytogenes* invades the human body thanks to the ability of crossing the gastrointestinal barrier by inducing its own endocytosis in epithelial cells. Upon crossing of the cell membrane, *L. monocytogenes* replicates until it spreads outside the cell to infect the neighboring environment. The infection typically gets a broad diffusion through the host body when bacteria reach the bloodstream [6].

Clinical manifestations of *L. monocytogenes* infection come as a consequence of multiple events; however, both adhesion and cell invasion are key factors in host susceptibility and in determining the diverse capability of the various *L. monocytogenes* strains to invade the host cells [7]. Specifically, internalins, a group of *L. monocytogenes* surface proteins, have shown to play a key role in mediating the cell invasion [7,8]. Even though more than 25 internalin genes have been identified, internalin A (*inlA*) is thought to have a pivotal role in cell invasion being relevant for cell surface anchorage and uptake induction by gastrointestinal epithelial cells [7,9]. Indeed, epidemiological studies described that the full length *inlA* gene was identified in most of clinical strains (above 95%) [10,11]. Conversely, strains holding nonsense mutations that result in premature stop codons (PMSCs) usually express a truncated gene product and, as a consequence, they typically show an attenuated virulence [12,13]. Thus far, 21 mutations leading to truncated variants have been identified [14].

The *inlA* gene product (InlA) is an 80 kDa protein containing 15 and a half leucine-rich repeats (LRRs), a signaling peptide at the N-terminal and an LPXTG bacterial surface-anchoring motif at the C-terminal [15]. The main host cell target of InlA is the surface protein E-cadherin (Ecad). In particular, the InlA LRRs recognize and bind the EC1 domain of the extracellular portion of Ecad, as documented by crystallographic studies [9]. Such a binding event is critical to initiate the molecular cascade leading to the internalization of *L. monocytogenes* by the host cells [15,16]. In this respect, several InlA mutated forms reducing the capability to interact with Ecad have been identified [17]. Among them, truncated InlA variants are typically described and their reduced capability to contact Ecad may provide a rationale to explain, at least in part, the attenuated virulence of certain *L. monocytogenes* strains [13,17]. However, the existence of missense mutations leading to amino acid substitutions was described too. Notably, *in vitro* studies showed that such mutations may either prevent the InlA-Ecad interaction or strongly promote their binding enhancing host cells invasion [9,18]. The influence of these mutations on *L. monocytogenes* virulence is therefore likely, though largely overlooked, and their characterization might result in a more aware comprehension of *L. monocytogenes* epidemiology. In this respect, the role of *inlA* as major virulence factor of *L. monocytogenes* has been largely investigated in the recent years and its pivotal role in determining the success of infection *in vivo* is getting more and more debated. In spite of the prevalence of full-length InlA sequences in clinical isolates, a growing number of evidences suggest no direct correlation between either *inlA* sequence or InlA integrity and the capability to invade cells or to cause infection [14,19]. This scenario has made urgent a better understanding of the molecular basis underlying InlA-Ecad interaction and the investigation of missense mutations deserves a particular interest being potentially important to finely modulate InlA-Ecad complex formation.

The work presented here is framed within the context of developing a computer-driven system analysis to timely identify, characterize and hierarchize for further analysis InlA missense mutations on the basis of their possible capability to modify the interaction with human Ecad. To note, in silico analysis already proved to be a reliable analytical tool to investigate the interaction of macromolecules with either small molecules (e.g., [20]) or other macromolecules, as shown already for InlA-Ecad [21]. Additionally, the computational assessment of inter-molecules interaction may eventually result in a reliable estimate of biological outcomes (e.g., [22,23,24,25]). Specifically, in the present work, a structure-based molecular modeling approach was developed and validated as a proof of concept using a set of previously characterized engineered mutations [9]. Then, the model was challenged with an InlA variant identified in food isolates to characterize for the first time its capability to interact with Ecad. Overall, the workflow presented here proved to be a reliable tool to study the molecular basis of InlA-Ecad interaction. Furthermore, it could be a promising platform of analysis to tackle the early study of missense mutations of other *L. monocytogenes* virulence factors to better support the molecular understanding of its epidemiology.

## 2. Results and Discussion

Mutations in the *inlA* gene may lead to protein variants with a diverse capability to promote cell invasion. While PMSCs are usually associated with an attenuated invasiveness, the effect of missense mutations on InlA-Ecad interaction, which may either result in lower or higher invasion capacity, can be an important factor to get dissected for a preliminary evaluation of either novel *L. monocytogenes* isolates or new and uncharacterized InlA variants. Therefore, in the context of providing a framework for the rapid and early identification of strains to be considered at risk, the present study aimed at checking whether a structure-based computational approach can reliably compute the effects of missense mutation in the InlA-Ecad complex formation. To do so, a set of four engineered InlA mutants previously described influencing InlA-Ecad complex formation was analyzed and compared to the wt InlA-Ecad complex. Specifically, the mutated InlA variants considered in this study hold the following mutation: Phe367Ala and Tyr343Ala, which both proved to significantly reduce the InlA-Ecad interaction [9], and Tyr369Ser and the double mutant Ser192Asn-Tyr369Ser, which proved to increase the InlA-Ecad complex formation [18]. The computational study relied on the calculation of possible effects of each substitution in terms of single-residue contribution to the InlA-Ecad interface interaction. Either the favors or impairments due to mutations at the protein-protein interface were assessed estimating the overall favors of interaction at the proteins contact interface using the HINT scoring function [26]. In addition, a pharmacophoric analysis of the space surrounding each mutation described such effects mechanistically. Specifically, the spatial distribution of the substituted amino acids in respect to the physicochemical properties of the space at the InlA-Ecad interface wherein they could arrange was considered. Subsequently, molecular dynamics simulations were run to investigate the effects of the set mutations under analysis on the geometric stability of InlA-Ecad complexes over the time. 

### 2.1. Assessing the Effects of Mutations on Interface Interaction

**Figure 1:**
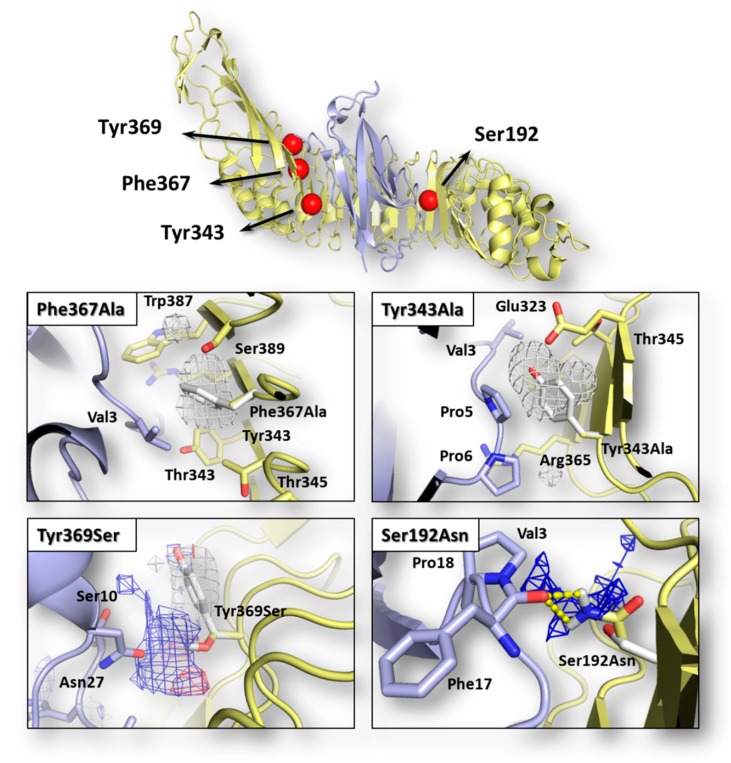
Graphical representation of InlA-Ecad complex and pharmacophorical analysis of the space surrounding mutations. Both InlA (yellow) and Ecad (light blue) are represented in cartoon, while the red spheres indicate the localization of mutations considered in this study. Amino acids relevant for interface interactions are represented in sticks (those belonging to the wt InlA-Ecad complex are white colored). The regions energetically and sterically suitable to receive hydrophobic or hydrophilic groups are represented in white or blue mesh, respectively, while yellow dotted lines indicate hydrogen bonds.

The possible effects of each mutation in terms of single-residue contribution to the InlA-Ecad interface interaction were investigated using the HINT scoring function as it previously succeeded to assess protein-protein complex formation and stability (e.g., [22]; see Section 2.3 for further details). As shown in Table 1, each InlA-Ecad interaction was qualitatively scored in accordance with the experimental data for all the complexes considered. As shown in Figure 1, the pharmacophoric analysis of the space surrounding each mutation provided a structural explanation to the diverse scores recorded by mutated variants in comparison to the wt complex. For both mutations reducing the InlA-Ecad interaction (i.e., Tyr343Ala and Phe367Ala), the amino acid substitution was found to cause a loss of favored hydrophobic/hydrophobic interaction, thereby explaining the reduction of scores. In particular, in the wt complex, Tyr343 and Phe367 were found arranging the respective side chain within a hydrophobic space at the InlA-Ecad interface reasonably adding a favorable contribution to protein-protein interaction. In both the mutated variants, the two amino acids were substituted with an Ala residue and its shorter side chain could not reach those hydrophobic regions failing to fulfill the aforementioned favorable contribution. In addition, the formation of ancillary InlA-Ecad interactions mediated by water molecules was thought not likely in those regions, as their stark hydrophobicity did not support a reasonably stable residence of water molecules. Conversely, with respect to the mutations enhancing InlA-Ecad interaction (i.e., Tyr369Ser and the double mutant Ser192Asn-Tyr369Ser), the amino acid substitutions were found adding favorable contributions to the interface interaction. In more detail, Ser192Asn mutation was found forming an additional hydrogen bond-mediated direct polar contact between InlA and Ecad. Concerning Tyr369Ser mutation, the substitution was found fulfilling a hydrophilic space facing the Ecad residue Asn27. In addition, even though the Ser369-Asn27 inter-residue distance and angle was not suitable to form hydrogen bonds, the formation of favorable acid-base interaction was observed. 

On this basis, the scoring of InlA-Ecad interaction coupled to the pharmacophoric analysis of space surrounding mutations at the interface proved to be a reliable qualitative assessment to distinguish which single missense mutations at the complex interface may result either in favored or impaired InlA-Ecad interactions in comparison to the wt complex.

### 2.2. Assessing the Effects of Mutations on the Geometrical Stability of Complex over the Time 

**Figure 2:**
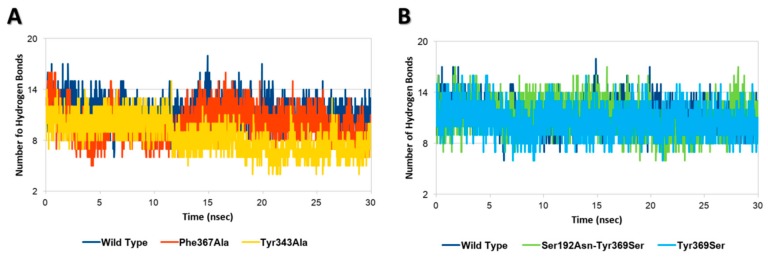
Hydrogen bonds analysis. (**A**) Comparison between wt InlA-Ecad complex and mutated variants impairing InlA-Ecad interaction. (**B**) Comparison between wt InlA-Ecad complex and mutated variants promoting InlA-Ecad interaction.

Each InlA-Ecad complex underwent molecular dynamic simulations to assess the possible effects of mutations on the geometrical stability of InlA-Ecad interaction over the time. The overall analysis of complex stability relied on the root-mean-square deviation (RMSD) analysis of protein C-alpha, while the root-mean-square fluctuation (RMSF) analysis of protein C-alpha was performed to locally measure the mobility of protein residues, in agreement with previous studies [27]. According to the results of RMSD and RMSF analysis, there were no appreciable differences among the mutated variants and the wt complex, as, in all the cases analyzed here, each complex kept stable its overall geometry regardless of the mutation hold. This evidence pointed to the overall stability of InlA and it was in agreement with other authors that were not able to show a geometrical destabilization of unfavorable InlA-Ecad complex in molecular dynamic simulations at a nanosecond scale [21]. Nonetheless, in the present study, the thorough analysis of InlA-Ecad interface contacts network revealed significant difference between destabilizing (Tyr343Ala and Phe367Ala) and stabilizing mutations (i.e., Tyr369Ser and the double mutant Ser192Asn-Tyr369Ser) that could be used to appreciably distinguish the qualitative effects of missense mutations. In more detail, the complexes holding mutations impairing the InlA-Ecad interaction recorded a reduction of the overall number of hydrogen bonds, which was more pronounced in the case of Tyr343Ala, in comparison to the wt complex (Figure 2A). Conversely, those variants that hold mutations increasing InlA-Ecad interaction scored a comparable or slightly higher number of hydrogen bonds than the wt complex (Figure 2B).

Moreover, the network of durable short-range contacts was analyzed to estimate the non-polar contributions to the InlA-Ecad interaction, in agreement with previous studies [28]. Interestingly, this analysis pinpointed a diverse network of interactions between the mutated variants and the wt complex. In particular, the wt complex recorded a total of 53 contacts between InlA and Ecad, while the mutated variants scored a total of 55 contacts and 56 contacts, in the case of Tyr369Ser and the double mutant Ser192Asn-Tyr369Ser, respectively. Conversely, both variants bearing mutations impairing the InlA-Ecad complex scored lower numbers with 51 contacts in the case of Phe367Ala and 48 contacts in the case of Tyr343Ala. 

**Figure 3:**
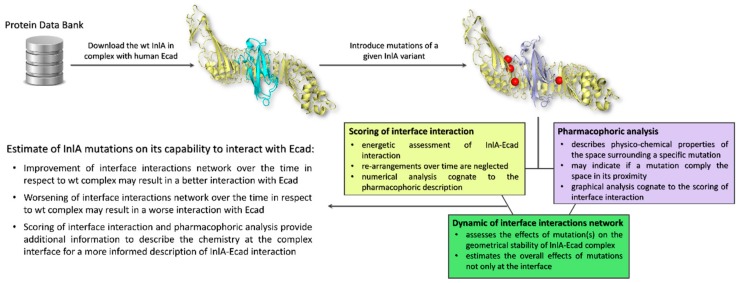
Schematic representation of the workflow used.

Taken together, these results highlighted that when mutations impaired the InlA-Ecad interaction, they were not compliant with the surrounding pharmacophoric space (see Section 4.2 for further details). In addition, they could also cause a more general reorganization at the complex interface resulting in a drop of hydrogen bonds and short-range contacts. Conversely, those mutations that caused an increase of InlA-Ecad interaction were found adding pharmacophoric favors at the interface interaction (see Section 4.2 for further details) and increasing the overall number of hydrogen bonds and short-range contacts. From a general point of view, the scoring of interface interaction and the pharmacophoric analysis have to be integrated to each other (thereby describing the chemistry at the complex interface) and to the molecular dynamics outcome to provide an informed and comprehensive evaluation of the InlA-Ecad complex stability (the overall workflow applied is reported in Figure 3). Therefore, in the eventuality given InlA variants provide ambiguous and similar results in terms of interactions network at the InlA-Ecad interface, they could not be appreciably distinguished and their capability to interact with Ecad should be considered comparable.

Therefore, based on the above, specific pharmacophorical, energetic and geometrical indicators were consistently identified, and their combined use succeeded to provide a qualitative assessment of the possible effects of missense mutations of InlA on its interaction with Ecad. In addition, keeping in mind that the complex dissociation in the case of unstable complexes was not observed at the nanoseconds scale [21], the change of interface interactions network could be regarded as the early and causal molecular mechanism leading to the complex detachment on a later stage.

### 2.3. Assessing the Effects of an InlA Variant from Food Isolates on InlA-Ecad Interaction 

**Figure 4:**
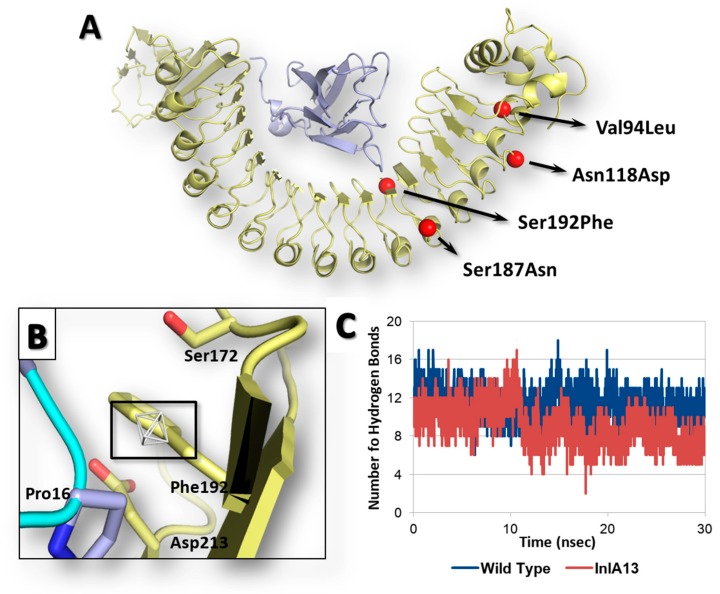
Results of InlA13 variant. Both InlA (yellow) and Ecad (light blue) are represented in cartoon, while amino acids side chains relevant for interface interactions are represented in sticks. (**A**) Graphical representation of InlA13 variant. (**B**) Close-up of Ser192Phe substitution at the InlA-Ecad interface. The region energetically and sterically suitable to receive hydrophobic groups is represented in white mesh and black box. (**C**) Comparative hydrogen bond analysis of InlA13-Ecad and wt InlA-Ecad complexes.

Once the reliability of computational workflow was proved, an InlA variant identified in food isolates retrieved from the Listeria Sequence Typing repository (https://bigsdb.pasteur.fr) [29] never characterized before in terms of interaction with Ecad was investigated, as a proof of principle, to characterize the possible effects of its mutations on the capability to interact with Ecad. Specifically, the InlA variant under analysis (InlA13) (locus *inlA* (lmo0433), allele 13; according to the Listeria Sequence Typing repository classification) had the following missense mutations and no PMSCs: Val94Leu, Asn118Asp, Ser187Asn and Ser192Phe (further information are reported in Supplementary Materials Figures S1 and S2). Of note, the position 192, which is at the InlA-Ecad interface (Figure 4A), was already considered for missense mutagenesis, and its substitution with an Asn resulted in an enhanced InlA-Ecad interaction (see above).

The calculation of interface interaction showed potentially improving effects due to Ser192Phe mutation as the HINT score recorded was 4489 units, which is slightly higher (1%) than the interaction scored by the wt InlA-Ecad complex (4464 units). The close inspection of pharmacophoric requirements at the InlA-Ecad interface could explain the slight score increase as the side chain of Phe192 was arrange close to a small region of the space at the InlA-Ecad interface able to receive hydrophobic group (Figure 4B). Nevertheless, the limited extension of the hydrophobic region with respect to the volume of Phe side chain did not suggest a fully satisfying match, in agreement with the very slight score increase observed. Moreover, the InlA13-Ecad complex underwent molecular dynamic simulations to check its geometric stability over the time. As observed for the other cases (see above), the complex was found stable in terms of C-alpha RMSD and RMSF analysis. However, the analysis of interactions network at the InlA13-Ecad showed an overall marked reduction of contacts number in comparison to the wt complex reflecting the overall impairing effects of the mutations hold by InlA13. Indeed, the number of hydrogen bonds experienced an early and marked drop over the time with respect to the wt complex (Figure 4C). Additionally, the number of short-range contacts observed was 1 unit lower than in the case of wt complex (52 contacts instead of 53).

On the basis of these results, the whole set of mutations of InlA13 were overall calculated to impair the interaction with Ecad. In spite of the calculated capability of Ser192Phe to slightly favor the interface interaction, Val94Leu, Asn118Asp and Ser187Asn were computed having an adverse effect on the overall capacity of InlA13 to promote a stable and favored network of interactions at the complex interface over the time. Taken together, these results further confirmed the complementarity of interface interaction scores obtained using HINT, pharmacophoric description and data from MD simulations for a detailed description of both the chemistry and dynamic aspects of the network of interactions at the InlA-Ecad interface. Indeed, amino acid substitution distal from the contact interface might either result in a worse (as in this case) or improved network of interactions in time, regardless of the individual contribution of mutations at the interface.

Notably, the sequence of InlA13 investigated in the present work was encoded in a series of *L. monocytogenes* isolates (e.g., GeneBank accession codes APID00000000 and AWWR00000000) previously assessed for their capability to invade cells [14]. In particular, they showed a mean cell invasion capacity either nearly 1 log CFU higher (as in the case of strain LM438, AWWR00000000) or lower (as in the case of strain SHL004, APID00000000) than the reference EGD-e strain (holding the wt InlA sequence analyzed in the present work). On the one hand, our results may provide a mechanistic and InlA-dependent rationale explaining the reduced invasion capacity observed for the strain SHL004 (APID00000000), though they failed to explain those strains with a raised invasiveness [14]. On the other hand, our results pointed out that the strength of InlA-Ecad interaction may not directly relate to the cell invasion capacity reflecting at the same time the possible non-exclusive role of InlA in determining the virulence of *L. monocytogenes*, in agreement with previous studies [14,17].

## 3. Conclusions

Our work showed a cost-effective and time-saving framework of analysis to investigate the potential effects of *inlA* missense mutations on the very early molecular event underlying the cell invasion of *L. monocytogenes*. In more detail, our work proved for the first time that a molecular modeling study may succeed to qualitatively calculate the effects of *inlA* missense mutations in relation to the capability to stabilize InlA-Ecad complex. Of note, in case of simultaneous mutations at the InlA-Ecad interface and in other positions not in its proximity, the scoring of effects of mutations at the interface has to be integrated by MD simulations to thoroughly calculate the global effects on the InlA-Ecad complex stability over the time.

Overall, the approach proposed described a mechanistic rationale to explain both impairing and promoting effects of mutations, providing also a valuable tool to analyze InlA variants found in real isolates. In this respect, the analysis of an InlA variant (InlA13) from a food *L. monocytogenes* isolate was performed and impairing effects of its mutations on the capability to contact Ecad were observed. This evidence, in the light of experimental evidences collected previously, pointed out that a strong InlA-Ecad interaction might not be essential to determine the cell invasion capacity of *L. monocytogenes*. Therefore, our results are in line with the finding that InlA has an important, though not exclusive, role in determining the virulence of *L. monocytogenes* strains, as previously suggested [13]. Nevertheless, the role of *inlA* missense mutations needs to be dissected precisely to better understand the mechanisms of *L. monocytogenes* virulence and the workflow presented can be an effective analytical approach. In this respect, a systematic application of the paradigm of analysis proposed may eventually improve the characterization of *L. monocytogenes* isolates. In addition, it may support surveillance plans eventually identifying isolates of particular concern in relation to their potential capability to strongly interact with Ecad. Moreover, the method proposed can also be theoretically applied to other virulence factors to foster a broad characterization of *L. monocytogenes* virulence from a chemical and molecular stand point. Among them, and in line with the assessment of early mechanisms of infection, listeriolysin O shall have a high priority being found as an important cooperative determinant to increase the efficiency of host cell invasion [30].

## 4. Materials and Methods 

### 4.1. Molecular Modeling

The three-dimensional (3D) model of InlA-Ecad complex (Figure 1) was derived from the crystallographic coordinates of the wild type (wt) complex recorded in the Protein Data Bank (PDB; https://www.rcsb.org) [31] with PDB code 1O6S [9]. The complex consists of the *L. monocytogenes* EGD-e’s InlA (residues 36-495) and the wt human Ecad immunoglobulin-like domain 1 (residues 4-98). The complex structure was processed using the Sybyl software, version 8.1 (www.certara.com) checking the consistency of atom and bond types assignment and removing the co-crystalized low-molecular weight molecules, as previously reported [32]. Mutated variants (see Section 2 for further details) were obtained from the wt model introducing mutations with the “Mutate Monomers” option in the “Biopolymer” module of Sybyl, version 8.1 (www.certara.com). A mild local minimization of each mutated residue (Powell algorithm with 250 iterations or 0.05 kcal/(mol∙Å) as computation thresholds) was done to avoid improper atomic coordinates arrangement, in agreement with previous studies [33]. The graphics were acquired using The PyMol Molecular Graphic System, Version 1.8.4 Schrödinger, LLC. (https://sourceforge.net/projects/pymol/files/pymol/1.8/).

### 4.2. Assessment of Interface Interaction

The capability of each mutation to affect the InlA-Ecad surface interaction was assessed and compared to the wt complex computing the overall interaction score of each complex with the HINT scoring function [26]. In particular, HINT score has an inverse relationship to the free energy of binding (the higher the score, the stronger the interaction expected) [34] and it previously proved reliable to compute protein-macromolecule complex formation (including protein-protein and protein-DNA complex) [22,35].

### 4.3. Pharmacophoric Modeling

The physico-chemical space surrounding each mutated residue was described to provide a visual explanation of the effects of mutations on the InlA-Ecad interface interaction. The extension of chemical space to be analyzed was defined using the Flapsite tool of the FLAP software (www.moldiscovery.com/software/flap), while the GRID algorithm was used to investigate the corresponding pharmacophoric fingerprint [36,37]. In more detail, each pocket search was done by residue, selecting each mutated residue and setting the pocket extension and thickness at 6 and 5, respectively. The DRY probe was used to describe potential hydrophobic interactions, while the sp2 carbonyl oxygen (O) and the neutral flat amino (N1) probes were used to describe the hydrogen bond acceptor and donor capacity of the target, respectively.

### 4.4. Molecular Dynamic Simulations

Molecular dynamic (MD) simulations were performed to study the dynamic of interactions of each complex over the time using GROMACS (version 5.1.4; www.gromacs.org) [38] with CHARMM27 all-atom force field parameters support [39], in agreement with a previous study [33]. Briefly, each complex was solvated with SPCE waters in a cubic periodic boundary condition, and counter ions (Na^+^ and Cl^−^) were added to neutralize the system. Prior to MD simulation, the systems were energetically minimized to avoid steric clashes and to correct improper geometries using the steepest descent algorithm with a maximum of 5000 steps. Afterwards, all the systems underwent isothermal (300 K, coupling time 2 psec) and isobaric (1 bar, coupling time 2 psec) 100 psec simulations before running 30 nsec simulations (300 K with a coupling time of 0.1 psec and 1 bar with a coupling time of 2.0 psec). Then, a residue-residue interface analysis along each simulation was done to describe the pattern of long-lasting short-range interatomic interaction between InlA and Ecad using g_contacts software [40]. In agreement with previous studies, the analysis could be used to approximate the non-polar contributions to the intermolecular interaction [28,41]. In particular, the cutoff frequency for long-lasting contacts was set at 0.4 to identify those that occurred in at least 40% of the simulation. 

## Figures and Tables

**Table 1:** Computational scores of wt and mutated InlA-Ecad interface interactions.

InlA Variant	Experimental Evidence ^a^	HINT Score ^b^	% Variation ^c^
Wild type (*L. monocytogenes* EGD-e)	---	4464	---
Phe367Ala	↓	4387	−2%
Tyr343Ala	↓	4321	−3%
Tyr369Ser	↑	4596	+3%
Ser192Asn-Tyr369Ser	↑	4650	+4%

Note: ^a^ promoting or impairing effects on InlA-Ecad complex formation of InlA mutations in comparison to the wt are indicated by ↑ and ↓, respectively, according to [9] and [18]; ^b^ HINT scores inversely correlate to the free energy of binding (i.e., the higher the score, the stronger the interaction), and therefore, scores higher or lower than the wt complex may indicate more or less favored interaction, respectively; ^c^ the percentage variation in comparison to the wt is reported.

## Supplementary Materials

The following are available online at https://www.mdpi.com/2072-6651/12/1/60/s1, Figure S1: DNA sequence of InlA variant under analysis (locus lmo0433, allele 13; according to Listeria Sequence Typing Repository classification), Figure S2: Primary protein sequence of InlA13 (mutations in respect to the wt EGD-e protein are highlighted in red).
