# Supplementary material for: A Structural Study on the Listeria Monocytogenes Internalin A—Human E-cadherin Interaction: A Molecular Tool to Investigate the Effects of Missense Mutations
Source: Toxins (Basel). 2020 Jan 20;12(1):60. doi: 10.3390/toxins12010060 (PMC7020427; doi:10.3390/toxins12010060)
Supplement: Supplementary file 1 [file toxins-12-00060-s001.pdf]

Toxins **2020**, *12*, 60; doi:10.3390/toxins12010060

Supplementary Materials: A Structural Study on the *Listeria Monocytogenes* Internalin A - Human E-cadherin Interaction: A Molecular Tool to Investigate the Effects of Missense Mutations

Luca Dellafiora, Virginia Filipello, Chiara Dall'Asta, Guido Finazzi, Gianni Galaverna and Marina Nadia Losio

```
GTGAGAAAAA AACGATATGT ATGGTTGAAA AGTATACTAG TAGCAATATT AGTATTTGGC
AGCGGAGTAT GGATTAACAC GAGTAACGGG ACAAATGCTC AGGCAGCTAC AATTACACAA
GATACTCCTA TTAATCAGAT TTTTACAGAT ACAGCTCTAG CGGAAAAAAT GAAGACGGTC
TTAGGAAAAA CGAATGTAAC AGACACGGTC TCACAAACAG ATCTAGACCA AGTTACAACG
CTTCAGGCGG ATAGATTAGG GATAAAATCT ATCGATGGAT TGGAATACTT GAACAATTTA
ACACAAATAA ATTTCAGCAA TAATCAACTT ACGGATATAA CGCCACTTAA AGATTTAACT
AAGTTAGTTG ATATTTTGAT GAATAATAAT CAAATAGCAG ATATAACTCC GCTAGCTAAT
TTGACGAATC TAACTGGTTT GACTTTGTTC AACAATCAGA TAACAGATAT AGACCCGCTT
AAAAATCTAA CAAATTTAAA TCGGCTAGAA CTATCTAGTA ACACGATTAG TGATATTAGT
GCGCTTTCAG GTTTAACTAA TCTACAGCAA TTATTTTTTG GTAATCAAGT GACAGATTTA
AAACCATTAG CTAATTTAAC AACACTAGAA CGACTAGATA TTTCAAGTAA TAAGGTGTCA
GATATTAGTG TTCTGGCTAA ATTAACCAAT TTAGAAAGTC TTATCGCTAC TAACAACCAA
ATAAGTGATA TAACTCCACT TGGGATTTTA ACAAATTTGG ACGAATTATC CTTAAATGGT
AACCAGTTAA AAGATATAGG CACATTGGCG AGTTTAACAA ACCTTACAGA TTTAGATTTA
GCAAATAACC AAATTAGTAA TCTAGCACCA CTGTCGGGTC TAACAAAACT AACTGAGTTA
AAACTTGGAG CTAACCAAAT AAGTAACATC AGTCCCCTAG CAGGTTTAAC CGCACTCACT
AACTTAGAGC TTAATGAAAA TCAGCTGGAA GATATTAGCC CAATTTCTAA CCTGAAAAAT
CTCACATATT TAACGTTGTA CTTTAATAAT ATAAGTGATA TAAGCCCAGT TTCTAGTTTA
ACAAAGCTTC AAAGATTATT TTTCTATAAT AACAAGGTAA GTGACGTAAG CTCACTTGCG
AACTTAACCA ATATTAATTG GCTTTCGGCT GGGCATAACC AAATTAGCGA TCTTACACCA
TTGGCTAATT TAACAAGAAT CACCCAACTA GGGTTGAATG ATCAAGCATG GACAAATGCA
CCAGTAAACT ACAAAGCAAA TGTATCCATT CCAAACACGG TGAAAAATGT GACTGGCGCT
TTAATTGCAC CAGCTACTAT TAGCGATGGC GGTAGTTACA CAGAGCCTGA TATAACATGG
AACTTACCTA GTTATACAAA TGAAGTAAGC TATACCTTTA GCCAACCTGT CACTATTGGA
AAAGGAACGA CAACATTTAG TGGAACCGTG ACGCAGCCAC TTAAGGCAAT TTTTAATGCT
AAGTTTCATG TGGACGGCAA AGAAACAACC AAAGAAGTGG AAGCTGGGAA TTTATTGACT
GAACCAGCTA AGCCCGTAAA AGAAGGTCAC ACATTTGTTG GTTGGTTTGA TGCCCAAACA
GGCGGAACTA AATGGAATTT CAGTACGGAT AAAATGCCGA CAAATGACAT CAATTTATAT
GCACAATTTA GTATTAACAG CTACACAGCA ACCTTTGATA ATGACGGTGT AACAACATCT
CAAACAGTAG ATTATCAAGG CTTGTTACAA GAACCTACAC CACCAACAAA AGAAGGTTAT
ACTTTTAAAG GCTGGTATGA CGCAAAAACT GGCGGTGACA AGTGGGATTT TGCAACTAGT
AAGATGCCTG CTAAAAACAT CACCTTATAT GCTCAATATA GCGCCAATAG CTATACAGCA
ACCTTTGATG TTGATGGAAA AACAACGACT CAAGCAGTAG ACTATCAAGG ACTTCTAAAA
GAACCAAAAA CGCCAACAAA AGCCGGATAT ACTTTCAAAG GTTGGTATGA CGAAAAAACA
GATGGTAAAA AATGGGATTT TGCGACAGAT AAAATGCCAG CAAATGATAT TACGCTGTAC
GCTCAATTCA CGAAAAATCC TGTGGCACCA CCAACAACTG GAGGGAACAC GCCGCCGACT
ACAAATAACG GAGGGAATAC TACACCACCT TCCGCAAATA TACCTGGAAG CGACACATCT
AACACATCAA CTGGGAATTC AGCTAGCACA ACAAGTACAA TGAACGCTTA TGACCCTTAT
AATTCAAAAG AAGCTTCACT CCCTACAACT GGCGATAGCG ATAATGCGCT CTACCTTTTG
TTAGGGTTAT TAGCAGTAGG AACTGCAATG GCTCTTACTA AAAAAGCACG TGCTAGTAAA
TAG
```

Figure S1. DNA sequence of the InlA variant under analysis (locus lmo0433, allele 13; according to the Listeria Sequence Typing Repository classification).

```
>InlA13
GPLGSATITQDTPINQIFTDTALAEKMKTVLGKTNVTDTVSQTDLDQVTTLQADRLGIKSIDGLEYLNNLTQI
NFSNNQLTDITPLKDLTKLVDILMNNNQIADITPLANLTNLTGLTLFNNQITDIDPLKNLTNLNRLELSSNTI
SDISALSGLTNLQQLFFGNQVTDLKPLANLTTLERLDISSNKVSDISVLAKLTNLESLIATNNQISDITPLGI
LTNLDELSLNGNQLKDIGTLASLTNLTDLDLANNQISNLAPLSGLTKLTELKLGANQISNISPLAGLTALTNL
ELNENQLEDISPISNLKNLTYLTLYFNNISDISPVSSLTKLQRLFFYNNKVSDVSSLANLTNINWLSAGHNQI
SDLTPLANLTRITQLGLNDQAWTNAPVNYKANVSIPNTVKNVTGALIAPATISDGGSYTEPDITWNLPSYTNE
VSYTFSQPVTIGKGTTTFSGTVTQPLKA
```

Figure S2. Primary protein sequence of InlA13 (mutations in respect to the wt EGD-e protein are highlighted in red)
